# Beyond Pneumonia: A Rare Case of Pericardial Empyema Caused by Streptococcus pneumoniae

**DOI:** 10.7759/cureus.40450

**Published:** 2023-06-15

**Authors:** Harish Ashok, Reginald Manuel, Bushra Ahmed, Roy Lim, Reejeen Monsalve

**Affiliations:** 1 Internal Medicine, Ross University School of Medicine, Barbados, USA; 2 Internal Medicine, Mount Sinai Hospital, Chicago, USA; 3 Internal Medicine, Our Lady of Fatima University, Valenzeula, Metro Manila, PHL

**Keywords:** bacterial pericarditis, landmark-guided pericardiocentesis, recurrent pericardial effusion, streptococcus pneumonaie, internal medicine-cardiology, septic arthrits, purulent pericarditis

## Abstract

Purulent pericardial effusion is a rare but potentially deadly condition that demands immediate medical attention. When left untreated, it can have catastrophic consequences. While bacterial infection is the most common cause of this condition, it usually occurs in individuals with weakened immune systems or in those undergoing dialysis or thoracic surgery. This case report presented here is unique as it chronicles the uncommon experience of a 58-year-old male with a normally functioning immune system who suffered from purulent pericardial effusion, endocarditis, and pneumonia, all linked to septic arthritis of his knee caused by *Streptococcus pneumoniae*. The diagnosis and management of this condition require a swift and comprehensive approach, and any delay in treatment can have dire outcomes. This case highlights the significance of early detection and prompt treatment of purulent pericardial effusion to prevent severe complications and improve patient prognosis.

## Introduction

Pericarditis is a condition marked by inflammation of the pericardium, a thin sac that encloses and protects the heart. It is most often triggered by viral infections, with Coxsackie B, echovirus, and adenovirus being among the most common offenders [1]. However, bacterial infections can also lead to pericarditis, albeit less frequently, especially in individuals with a compromised immune system or in those who have recently undergone thoracic surgery. Among bacterial infections, *Streptococcus pneumoniae* is a well-known cause of purulent pericardial effusions, often spreading through direct contact [2]. In some cases, it can also spread hematogenously from distant sites and cause accompanying conditions such as pneumonia, subsequent empyema, and recurrent pericardial effusions [2]. Diagnosing bacterial pericarditis can be challenging as it may not always present with typical symptoms like fever, which may lead to delays in starting appropriate treatment [2]. While pericarditis often resolves on its own, symptomatic management may be necessary to relieve pain and discomfort [1].

In this report, we present an intriguing case of a 58-year-old man who, despite being immunocompetent, developed purulent pericardial effusion, myopericarditis, and pneumonia, all originating from a primary *S. pneumoniae* infection in his knee. This case underscores the importance of a comprehensive approach to the diagnosis and treatment of this condition, as any delay can have devastating consequences. It highlights the significance of early detection and management of purulent pericardial effusion to prevent severe complications and improve patient outcomes. Moreover, it emphasizes the need for clinicians to remain vigilant and consider atypical sources of infection, even in individuals who appear healthy.

## Case presentation

A 58-year-old male with a medical history of hypertension, non-insulin-dependent diabetes mellitus, and hypothyroidism, presented to the emergency department with chest pain and shortness of breath. The patient had been experiencing the symptoms for a week, which were described as intermittent, non-radiating, 7/10 in intensity, and accompanied by a productive cough and orthopnea. Additionally, he reported difficulty walking due to a recent onset of swelling and pain in his left knee. He denied associated fever, chills, any recent trauma, or IV drug use that might explain his symptoms. 

In the emergency department, he was found to be afebrile, having a blood pressure of 109/80 mmHg, tachycardic in the 120 s, and was discovered to be severely hypoxic, with a saturation level of only 50% on room air. Once placed on BiPAP 60%, his oxygen saturation improved considerably, reaching 99%. His significant laboratory results are shown in the table below (Table 1).

**Table 1:** Significant initial laboratory panel. mEq/L, milliequivalents per liter; mmol/L, millimoles per liter; pg/mL, picograms per milliliter; ng/mL, nanograms per milliliter; BUN, blood urea nitrogen

Laboratory parameters	Lab value	Reference range
White blood cell count	16.8 th/mm3	4.0-11.0
Hemoglobin	13.3 g/dL	12.0-16.0
BUN	67 mg/dL	7-25
Creatinine	2.8 mg/dL	0.50-1.10
Anion gap	33 mmol/L	4-11
Lactic acid	4 mmol/L	0.5-2.0
C-reactive protein	39.5 mg/L	0.0-0.9
Troponin I high sensitivity	1160 pg/mL	0-20
Brain natriuretic peptide	169 pg/mL	1-100
Sodium	136 mEq/L	136-145
Potassium	3.9 mEq/L	3.5-5.2
Chloride	91 mEq/L	98-107
Glucose	324 mg/dL	70-99
Hemoglobin A1c	8.0%	<5.7%
Beta-hydroxybutyrate	>4.5 mmol/L	0.1-0.3

**Figure 1:**
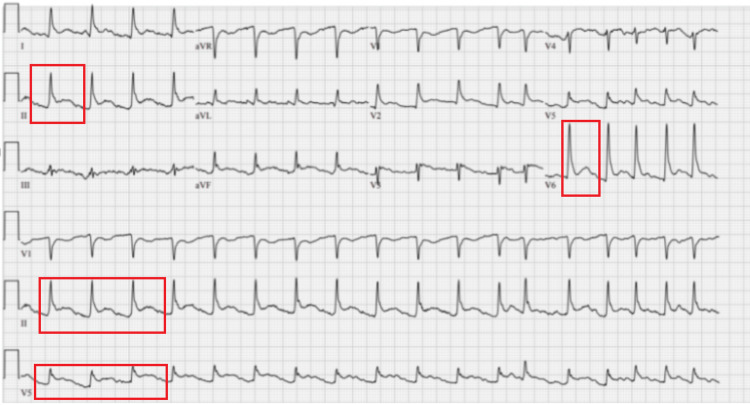
EKG demonstrating diffuse ST Elevations (red boxes). EKG, electrocardiogram

**Figure 2:**
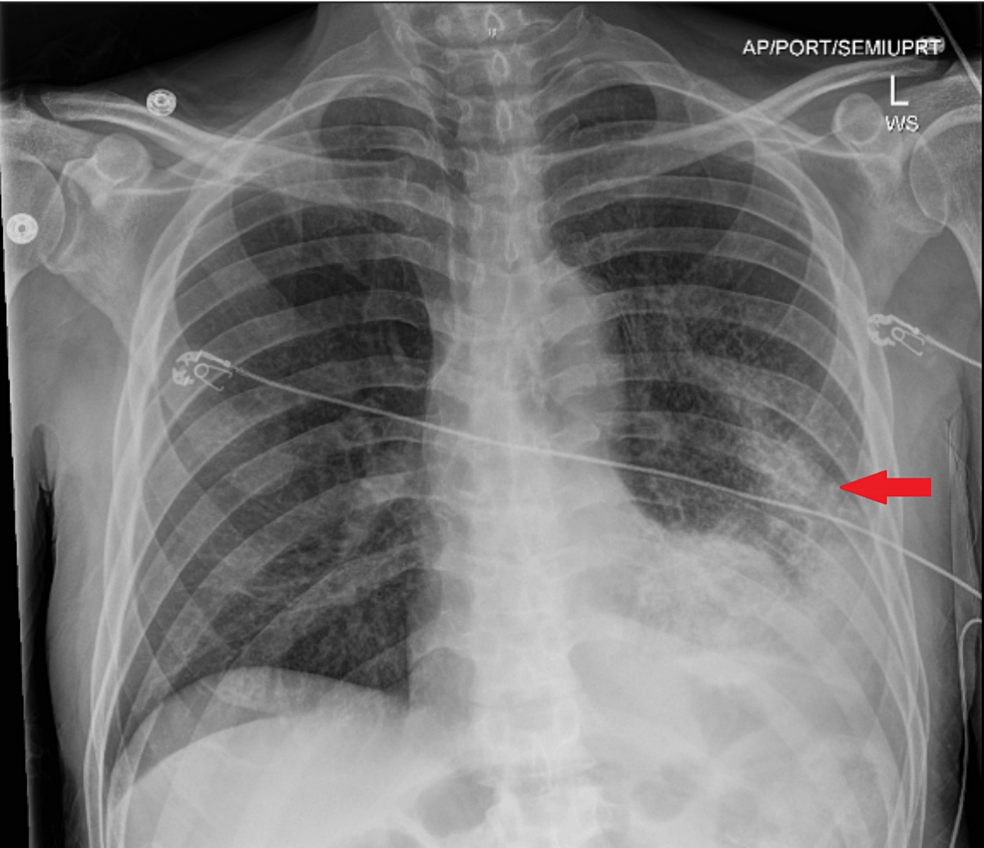
Chest X-ray (AP view) demonstrating large peribronchial infiltrates, with a moderate effusion at the left lower base (red arrow). AP, anterior posterior

The electrocardiogram revealed ST elevations in multiple leads, which raised concerns for pericarditis (Figure 1). The chest X-ray demonstrated large peribronchial infiltrates, with a moderate effusion at the left lower base (Figure 2). Preliminary blood cultures and left knee aspirate grew Gram positive cocci in chains and the patient was started on a course of IV Cefepime 1 g q8, Metronidazole 500 mg q8, and Vancomycin 1.5 g QD for two days. 

**Figure 3:**
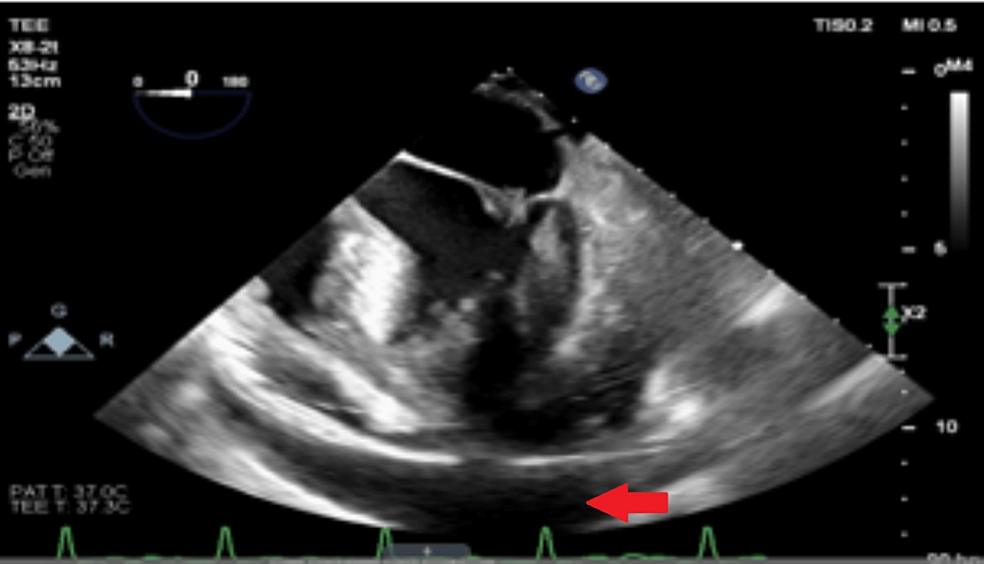
Echocardiogram demonstrating pericardial effusion (red arrow).

**Figure 4:**
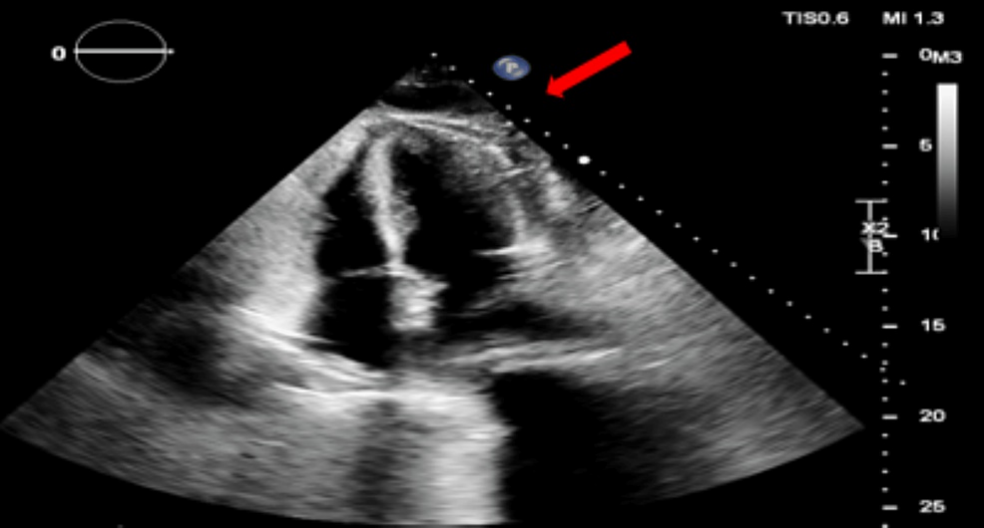
Echocardiogram: post pericardiocentesis and pericardial drain removal demonstrating recurrent pericardial effusion (red arrow).

Upon admission, an echocardiogram revealed moderate pericardial effusions on the left side and right ventricular free wall. Subsequent surveillance echocardiograms showed growing signs of hemodynamic compromise (Figure 3). Following a scheduled pericardiocentesis and pericardial drain placement, moderate purulent fluid with numerous white blood cells was obtained, with a daily drain output ranging from 50 to 200 cc over 10 days. After the removal of the drain, a limited echocardiogram revealed the continued presence of small, free-flowing pericardial effusions without hemodynamic compromise (Figure 4). 

As *S. pneumoniae* was identified in both blood cultures and the aspirate from the patient’s left knee, the antibiotic therapy was changed to IV Ceftriaxone 2 g administered every 12 h. The patient received this treatment throughout his hospital stay, which lasted for 29 days.

**Figure 5:**
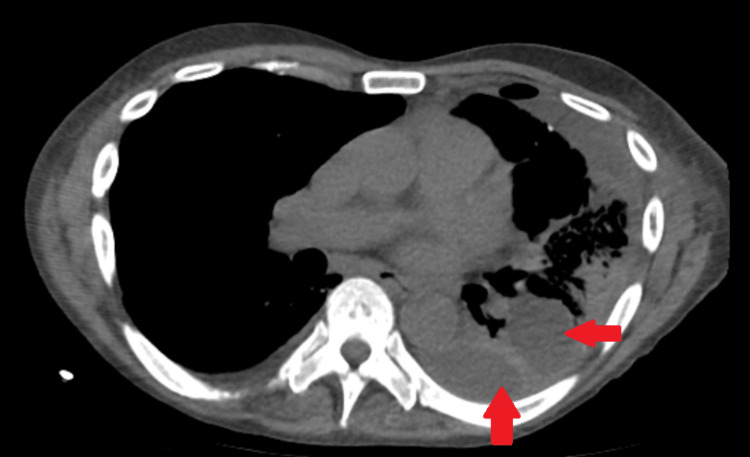
CT chest: multiloculated left-sided pleural effusions (red arrow).

Despite remaining afebrile and hemodynamically stable with some improvement in respiratory function, the patient experienced complications during his hospitalization. The surgical washout and debridement of his left knee necessitated the use of wound vac therapy for two weeks, while the CT scan of his chest revealed multiloculated left pleural effusions in multiple compartments, requiring pigtail drains for seven days (Figure 5). Though the patient demonstrated gradual improvement in respiratory function, the aforementioned complications added to the complexity of his treatment during his hospitalization.

A limited echocardiogram conducted before the patient’s discharge demonstrated the persistence of small free-flowing pericardial effusions without hemodynamic compromise. The patient was discharged with instructions to undergo an outpatient transthoracic echocardiogram within six weeks to monitor the progress of the effusions.

## Discussion

Purulent pericarditis, characterized by macroscopic or microscopic evidence of a pus-filled pericardial space, was once a common condition but has now become rare due to the introduction of antibiotics in the 1940s and more recently the pneumococcal conjugate vaccine. As a result, the incidence of bacterial endocarditis caused by *S. pneumoniae* has significantly decreased, currently estimated at 1 in 18,000 cases [3]. Although this condition was previously considered to affect mainly children and young adults, it is now observed in individuals as old as their fifth decade of life [4]. Despite being a rare complication of pneumonia, timely diagnosis and management are essential for a favorable prognosis. Notably, nearly half of the patients lack the classic signs and symptoms of fever, elevated central venous pressures, or pulsus-paradoxus [5].

Identifying bacterial pericarditis in a clinical setting can be challenging for several reasons. One major factor is the lack of typical physical indications, such as pericardial friction rub and pulsus paradoxus, which are observed in only about 30% of cases [2]. Furthermore, the clinical suspicion of this condition is often low due to its infrequent occurrence. Despite having a high mortality rate, bacterial pericarditis is not frequently diagnosed as antemortem due to its rapid progression and low incidence [2]. Therefore, healthcare providers should maintain a high degree of clinical suspicion for all patients, particularly those with risk factors such as immunocompromise and penetrating chest wall injuries. Delayed diagnosis and management can lead to complications such as tamponade, aneurysms, spread of infection, and pericardial constriction [5], which can result in a mortality rate approaching 40%, even with treatment [6]. 

Prompt investigation with echocardiography and pericardiocentesis is both diagnostic and therapeutic for bacterial pericarditis. Following the results of the pericardiocentesis, cultures of sputum, blood, or wound specimens can guide targeted antibiotic therapy [3]. Prolonged use of broad-spectrum antibiotics can extend hospitalization due to negative side effects, so it is important to monitor patient vitals, labs, and cultures to avoid overtreatment and associated complications. 

## Conclusions

*Streptococcus pneumoniae* is a multifaceted pathogen that can cause various uncommon manifestations beyond community-acquired pneumonia. It can affect different organ systems, such as joints and heart valves, by spreading through the bloodstream. In our patient's case, the infection reached the pericardium, resulting in purulent pericarditis, despite the absence of typical risk factors. The exact cause of this atypical condition remains unclear, highlighting the unpredictable nature of this infectious agent. Although the initial treatment was successful, the patient faced ongoing challenges, including recurrent effusions and pericardial fibrosis, demonstrating the unexpected and debilitating complications associated with this condition. Healthcare professionals need to be vigilant about severe complications and take appropriate measures to manage them as our understanding of bacterial purulent pericarditis continues to evolve. By increasing awareness and staying updated with the latest research, we can gain insights into its underlying mechanisms and develop more effective treatment strategies.
